# Supplementary material for: Assessment patient satisfaction towards emergency medical care and its determinants at Ayder comprehensive specialized hospital, Mekelle, Northern Ethiopia
Source: PLoS One. 2021 Jan 7;16(1):e0243764. doi: 10.1371/journal.pone.0243764 (PMC7790252; doi:10.1371/journal.pone.0243764)
Supplement: S1 File — (DOCX) [file pone.0243764.s002.docx]

[**S1 File.**](#_top)

**Questionnaire on assessing patient satisfaction towards the ED care**

1. **Sociodemographic characteristics of the patients**
2. What is your gender
3. Female
4. Male
5. Age_________ in years
6. What is your Level of education?
7. Illiterate
8. Read and write
9. Elementary
10. High School
11. Diploma
12. Degree and above
13. Please indicate your time of visit?
14. Morning
15. Evening
16. Night
17. Is it the first visit to this Hospital (frequency of visit)? ____
18. Who has completed the questionnaire
19. Patient
20. Another one
21. From where you came?
22. Urban
23. Rural
24. Waiting time before getting service __________in minute/hour
25. OPD site visited
26. Medical
27. Surgical
28. Pediatrics
29. Obstetrics/gynecology
30. Others
31. Subsequent decision made
32. discharged
33. admitted
34. operative theater
35. others specify__________
36. Degree of confidence to get good service in the future
37. Very confident
38. Confident
39. Somewhat confident

D. Not confident at all

12. Do you feel discriminated?

A. Yes

B. No

1. **Medical condition of the patients at emergency department**
2. Presence of any past illness
3. Yes
4. No
5. Emergency visit before
6. Yes
7. No
8. Number of emergency room visits ___________
9. Duration of stay in the emergency department ________ in hours
10. History of admission to the hospital
11. Yes
12. No
13. Previous chronic illness
14. Yes
15. No
16. If yes
17. High blood pressure
18. Cardiac problem
19. Cancer
20. Diabetes
21. Obesity asthma
22. Others __________________specify

| 1. **Brief Emergency Department Patients’ Satisfaction Scale (BEPSS)** | **1** | **2** | **3** | **4** | **5** |
| --- | --- | --- | --- | --- | --- |
| **Emergency department staff (EDS)** | | | | |  |
| 1. Nurses care about my treatment |  |  |  |  |  |
| 2. Nurses inform me about the remaining of the treatment |  |  |  |  |  |
| 3. Nurses attended to me patiently |  |  |  |  |  |
| 4. Nurses relieved me of the pain well |  |  |  |  |  |
| 5. Admission staff guided me appropriately |  |  |  |  |  |
| 6. The behavior of the admission staff was suitable |  |  |  |  |  |
| **Emergency department environment (EDE)** | | | | |  |
| 7. The environment of the emergency room was calm and quiet |  |  |  |  |  |
| 8. Emergency room was well equipped |  |  |  |  |  |
| 9. The environment of the emergency room was hygienic |  |  |  |  |  |
| **Physician care satisfaction (PCS)** | | | | |  |
| 10. The physician told me about my treatment course |  |  |  |  |  |
| 11. The behavior of the physician was respectful |  |  |  |  |  |
| 12. The physician’s explanation about the remaining of treatment was enough |  |  |  |  |  |
| 13. The physician spent a sufficient time examining me |  |  |  |  |  |
| **General patient satisfaction (GPS)** | | | | |  |
| 14. The waiting time before seeing the doctor was appropriate |  |  |  |  |  |
| 15. The waiting time before admission process was appropriate |  |  |  |  |  |
| 16. I would recommend this hospital to my acquaintances |  |  |  |  |  |
| 17. I am satisfied with the quality of services in the emergency room |  |  |  |  |  |
| 18. The emergency room of this hospital is well functioning |  |  |  |  |  |
| **Patient’s family satisfaction (PFS)** | | | | |  |
| 19. The family of the patient are respected in this hospital |  |  |  |  |  |
| 20. Family can spend an appropriate amount of time besides the patient |  |  |  |  |  |

**1-very dissatisfied, 2-dissatisfied, 3-fair/indifferent, 4-satisfied and 5-very satisfied.**
