# Supplementary material for: Assessment patient satisfaction towards emergency medical care and its determinants at Ayder comprehensive specialized hospital, Mekelle, Northern Ethiopia
Source: PLoS One. 2021 Jan 7;16(1):e0243764. doi: 10.1371/journal.pone.0243764 (PMC7790252; doi:10.1371/journal.pone.0243764)
Supplement: Attachment — Submitted filename: Response to reviewers.docx [file pone.0243764.s004.docx]

**Response to reviewers**

In general the comments and recommendations were accommodated as to the journal protocol and submission guidelines. Supplementary information were also attached and the concern raised with regards to the data availability, ethical committee concern, and copy right policy to replicate the tables used Table 4 (modified) and Table 5 (removed) were addressed. Our response to respective reviewers are stated below.

**Response to reviewer one:**

The tool has been pre-tested in Mekelle hospital Emergency department two weeks before the actual data collection and then tested for the internal reliability of the tool with Cronbach’s alpha result of 0.83.

**Response to reviewer two:**

No comment or question has been raised.

**Response to reviewer Three:** all your comments and recommendations are accepted and we have made an adjustment to the final manuscript. Table below here is the response to the questions raised.

| Questions | Response |
| --- | --- |
| 1. Most of the sentences are in passive voice; I would better prefer to use active voice especially in method section. like:"Every day, 200 patients visit the emergency department of the hospital" instead of "Daily it is visited by up to 200 patients." | We have changed some of the sentences that were written in passive voice. |
| 2. Why this sentence is in sample size calculation and sampling method? I am quite confused about it. "Busy work hours, different providers, day of the week and the type of client complaint were considered to affect satisfaction level." | The sentence has been moved from the sample size calculation section to the data collection procedure section. We considered it to affect patient satisfaction because in study conducted in Gondar they found the satisfaction to be affected by the day of the week visited, and busy work hour which was morning shift and another study also found that the type of complaint they present with could affect their satisfaction. So that considering these recommendations we tried to allocate the sample in each shifts, to get as diverse response as possible. |
| 3. Do avoid using didn't : instead use did not ( finding section) | Have been corrected |
| 4. I think interpretation of odds ratio is not appropriate: It should be like this: "The odds of patient who arrived during the morning time of the day, reporting to be satisfied were 4.8 times compared to their counterparts arriving at night ( AOR=4.8, 95% CI: 2.08, 11.4)." Do revisit interpretation of odds ratio minutely. | The interpretations of the odds ratio were also corrected as to your recommendation. |
| 5. You calculated Adjusted Odds ratio, but you did not mention what are the independent variables you adjusted for? Do mention clearly about it. | We added a sentence describing about the independent variables adjusted for in the result at the determinant of patient satisfaction section. |
| 6. I think it would be better to summarize your main finding in the first paragraph of the discussion section and then start to compare your findings with other studies. | The result were summarized in the first paragraph of the discussion. |
| 7. What might be the reason of variation in mean waiting time between your study and Iran's study? | The lengthy waiting time in the current study might be due to the burden of the high patient flow to the hospital since this is the only referral hospital with a wide catchment area. |
| 9. "This difference might be due to the difference in the tool used to measure patient satisfaction, difference in clinical characteristics (medical condition on arrival) of the patients and other different factors." In this sentence, other different factors means what, like? | It was a typing error we took it out and only difference in the tool used to measure patient satisfaction, and difference in clinical characteristics (medical condition on arrival) of the patients were stated as possible reasons. |
| 10. It would be better to discuss why patient visiting in morning time were more satisfied comparing with other studies. | We have included findings from other studies who also claimed that the satisfaction rate of patients was higher for those who visited the ED at the morning shift compared to the night shift. |
| 11. Why the study finding is contrary to the finding of study in Morroco in terms of educational status, as a predictor of patient  Satisfaction? | It was written as “contrary” which was not correct as its odds ratio showed the result was similar with that of Morroco and the reason have been justified. |

**Response to reviewer four:** all your comments and recommendations are accepted and we have made an adjustment to the final manuscript. Table below here is the response to the questions raised.

| Questions | Responses |
| --- | --- |
| 1. Please indicate the response rate of this study. | Response rate were stated in the result section of the manuscript which is 99.3%. |
| 2. A statement detailing including the reference number where appropriate of ethics committee, should appear in the manuscript. | The reference number for ethical review was provided in the manuscript. |
| 3. How are the reliability and validity of the questionnaire? | The questionnaire was translated both forward and backward then The internal consistency reliability was estimated with Cronbach’s α result of 0.83. |
| 4. Tables should also appropriately labeled to show the baseline results between respondents and non-respondents. | The tables were adjusted to state the non-response rate as (n=299) |
| 5. How the variables are selected in the logistic regression in Table 4? Please also show the goodness-of-fit results. | The variables on binary logistic regression with p-value <0.05 were subjected into multiple logistic regression. The Hosmer- Lemeshow goodness-of-fit result of (=0.737) was also presented at the Table 4. legend |
| 6. Please consider the comparison with the other epidemiological studies in other areas using table so make clear the significance of this study. | We tried to compare with study in Gondar northwest of Ethiopia, Hawasa in the southern of Ethiopia and Jimma in southwest Ethiopia. |
| 7. What is the originality and strengths of this study? How physicians or policy makers can deliberate with subjects based on the key findings of this paper? | Strength: this is the first study conducted in the Tigray region northern Ethiopia and would have a great input for clinicians and quality improvement of the hospital in terms of identifying potential areas of improvements and to plan strategies of the care provided at the ED accordingly. |
