# Supplementary material for: Assessment patient satisfaction towards emergency medical care and its determinants at Ayder comprehensive specialized hospital, Mekelle, Northern Ethiopia
Source: PLoS One. 2021 Jan 7;16(1):e0243764. doi: 10.1371/journal.pone.0243764 (PMC7790252; doi:10.1371/journal.pone.0243764)
Supplement: Attachment — Submitted filename: Reviewer comments PONE-D-20-06847.docx [file pone.0243764.s003.docx]

I have read this paper with great interest. The study assessed the degree of patient satisfaction regarding the Emergency Medical Services. The authors found the total patient satisfaction score towards emergency medical care to be good, as they also determined that the patient's waiting time is considered to be significantly associated with patient satisfaction level, as other research studies also identified waiting time in the clinic as an imporatant indicator of patient satisfaction. I did not identify any of major or minor concern. The research study has identified some important elements that are imperative to form the Emergency Medical Services organization.
